# The localization of Toll and Imd pathway and complement system components and their response to *Vibrio* infection in the nemertean *Lineus ruber*

**DOI:** 10.1186/s12915-022-01482-1

**Published:** 2023-01-12

**Authors:** Andrea Orús-Alcalde, Aina Børve, Andreas Hejnol

**Affiliations:** 1 Sars International Centre for Marine Molecular Biology, University of Bergen, Thormøhlensgate 55, 5008 Bergen, Norway; 2 Department of Biological Sciences, University of Bergen, Thormøhlensgate 53A, 5006 Bergen, Norway; 3 Faculty of Biological Sciences, Institute of Zoology and Evolutionary Research, Friedrich Schiller University Jena, Jena, Germany

**Keywords:** Toll pathway, Imd pathway, Complement system, Lectins, FreD-C, C-lectins, PRR, Nemertean, *Vibrio*, *Lineus*

## Abstract

**Background:**

Innate immunity is the first line of defense against pathogens. In animals, the Toll pathway, the Imd pathway, the complement system, and lectins are well-known mechanisms involved in innate immunity. Although these pathways and systems are well understood in vertebrates and arthropods, they are understudied in other invertebrates.

**Results:**

To shed light on immunity in the nemertean *Lineus ruber*, we performed a transcriptomic survey and identified the main components of the Toll pathway (e.g., *myD88*, *dorsal/dif/NFκB-p65*), the Imd pathway (e.g., *imd*, *relish/NFκB-p105/100*), the complement system (e.g., C3, *cfb*), and some lectins (FreD-Cs and C-lectins). In situ hybridization showed that *TLRβ1*, *TLRβ2*, and *imd* are expressed in the nervous system; the complement gene *C3-1* is expressed in the gut; and the lectins are expressed in the nervous system, the blood, and the gut. To reveal their potential role in defense mechanisms, we performed immune challenge experiments, in which *Lineus ruber* specimens were exposed to the gram-negative bacteria *Vibrio diazotrophicus.* Our results show the upregulation of specific components of the Toll pathway (*TLRα3, TLRβ1,* and *TLRβ2*), the complement system (*C3-1*), and lectins (*c-lectin2* and *fred-c5*).

**Conclusions:**

Therefore, similarly to what occurs in other invertebrates, our study shows that components of the Toll pathway, the complement system, and lectins are involved in the immune response in the nemertean *Lineus ruber*. The presence of these pathways and systems in *Lineus ruber*, but also in other spiralians; in ecdysozoans; and in deuterostomes suggests that these pathways and systems were involved in the immune response in the stem species of Bilateria.

**Supplementary Information:**

The online version contains supplementary material available at 10.1186/s12915-022-01482-1.

## Background

**Fig. 1:**
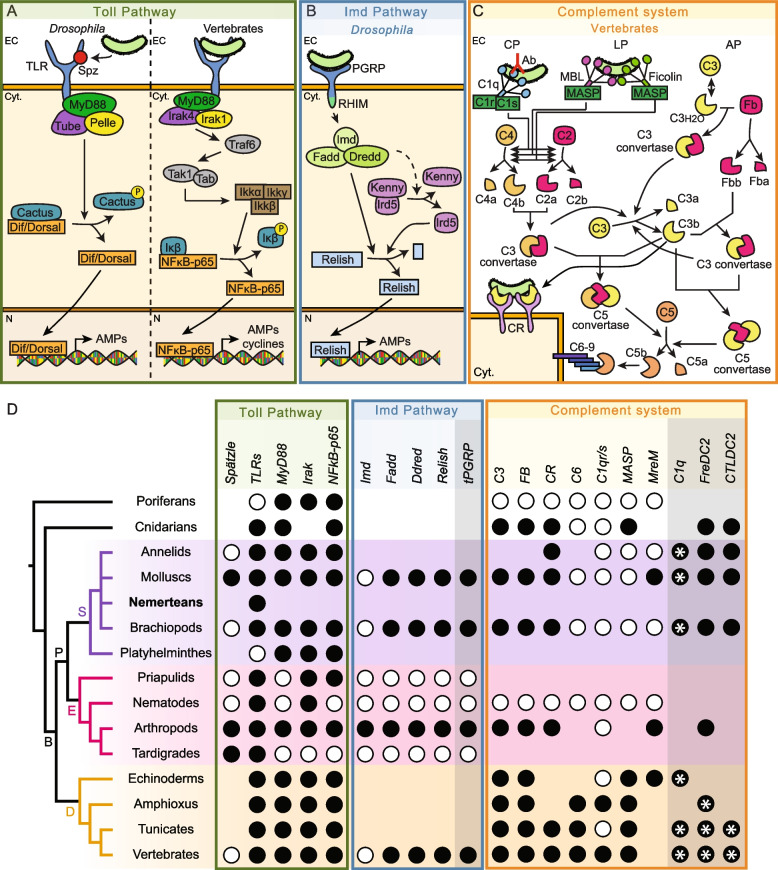
Toll pathway, Imd pathway, and complement system in metazoans. **A** Toll pathway, **B** Imd pathway, and **C.** complement system in *Drosophila* and vertebrates. Dashed arrows indicate indirect processes. **D** Presence and absence of proteins belonging to the Toll and the Imd pathways and to the complement system across metazoans. Orthologs of components of the Imd pathway have been found in vertebrates; however, these components belong to the vertebrate TNFα pathway, which is analogous to the arthropod Imd pathway. Grayish compartments within each pathway compartment indicate proteins that are uncertain to be involved in the pathway. Black circles indicate that the protein is present for that taxon, while white circles indicate its absence. For C1q, FreD-C, and C-lectin proteins, black circles with an asterisk (*) indicate the presence of proteins with collagen domains (C1qL, ficolin, and MBL/GBL, respectively), while only black circles indicate the presence of C1q, FreD-C, and C-lectin proteins containing coiled-coil regions instead of collagen domains (FreDC2 and CTLDC2). Nemertean phylogenetic position is highlighted in bold. AP, alternative pathway; B, Bilateria; CP, classical pathway; Cyt, cytoplasm; D, *Deuterostomia*; E, *Ecdysozoa*; EC, extracellular space; Fb, factor B; LP, lectin pathway; N, nucleus; P, *Protostomia*; S, *Spiralia*; tPGRPs: long transmembrane PGRPs. For references for **D**, view Additional file 1: Table S1. Phylogeny according to [6]

Innate immunity is the first line of defense of plants and animals against pathogens [1, 2]. During innate immunity, pathogen recognition receptors (PRR) can distinguish non-self from self by recognizing pathogen-associated molecular patterns (PAMP). In animals, PRRs are present in the main pathways and systems involved in innate immunity, such as the Toll and Imd pathways or the complement system [3–5] (Fig. 1).

The Toll pathway is an immune and developmental pathway present in many species across the metazoan tree [4, 7–9]. In vertebrates, the receptors of this pathway, named Toll receptors (TLRs), can recognize and bind PAMPs directly. However, in insects, PAMP recognition is mediated by the Spätzle protein (Fig. 1A). Once TLRs are activated, a signaling cascade, in which MyD88, Tube/Irak4, and Pelle/Irak1 are involved, triggers the entrance of NFκB transcription factors (Dorsal and Dif in *Drosophila* and NFκB-p65 in vertebrates) to the nucleus, which activate the expression of antimicrobial peptides and cytokines [8]. Besides vertebrates and *Drosophila*, components of the Toll pathway have been identified in multiple invertebrate species (Fig. 1D) [10–13]. Moreover, immune challenge assays have shown that the Toll pathway is involved in immunity in mollusks and crustaceans [14, 15]. Furthermore, the Toll pathway is also involved in a wide variety of developmental processes in many species of the metazoan tree [16–20].

The Imd pathway is involved in the arthropod immune response against bacteria [21–23]. Bacterial recognition triggers the activation of some long peptidoglycan recognition protein receptors (PGRP-Ls) [24]. Activation of these PGRP-Ls triggers a signaling cascade that includes the recruitment of the Imd, Fadd, and Dredd proteins and culminates with the entrance of the transcription factor Relish into the nucleus (Fig. 1B) [8]. The existence of this pathway outside arthropods is not clear. Components of this pathway are not present in other ecdysozoans, such as priapulids, nematodes, and tardigrades (Fig. 1D) [12]. Moreover, even though no homologous pathway to the Imd pathway has been identified in vertebrates, the Imd pathway shows similarities with the vertebrate TNF-α pathway, as orthologous proteins (e.g., Fadd, Dredd/Caspase8, Relish/NFκB) are present in both pathways [25]. However, the Imd protein is absent in vertebrates, and vertebrate PGRPs are not involved in TNF-α pathway activation. In spiralians, PGRPs and downstream components of this pathway are present in mollusks and brachiopods [11, 14, 26, 27]. However, PGRPs with RHIM motifs, essential for signal transduction in arthropod PGRPs, and the Imd adaptor have not been found in any of the two taxa.

The complement system is a proteolytic cascade involved in opsonization, phagocytosis, inflammatory regulation, and cytolytic processes. In vertebrates, this system is activated by three pathways: the classical, the lectin, and the alternative pathways (Fig. 1C) [28–30]. C1q is the receptor of the classical pathway, whereas the lectin pathway is activated by mannose-binding lectins (MBL) and ficolins [31–34]. These receptors trigger the activation of serine proteases (e.g., C1r, C1s, MASPs), which lead to the cleavage of the C3 protein. The alternative pathway is activated by the spontaneous hydrolysis of the C3 [29, 31–34]. C3 is the central component of the complement system, being the point where the three activating pathways converge [29]. The cleaved C3 protein can be detected by complement receptors (CR) present in phagocytic cells [35], but it can trigger the formation of the membrane attack complex (MAC) to induce cell lysis [36]. Although the complement system has been well studied in vertebrates, little is known about how this system functions in invertebrates. The core components of the complement system (C3, factor B, and complement receptors) are widespread through the metazoan tree (Fig. 1D) [27, 37–40]. Furthermore, *complement factor C* (*Cfc*) genes, which are homologous to *Cfb*, have been detected in some protostomes (e.g., arthropods, brachiopods) [27]. Moreover, while C1q proteins have been detected in spiralians [41], ficolins, MBL, and downstream proteins (e.g., C6) seem to be present in deuterostome invertebrates but not in protostomes [41–44]. However, C-lectins and fibrinogen-related domain-containing proteins (FreD-C) with similar domain architectures than ficolins and MBL have been identified in spiralians, suggesting that these proteins could perform analogous functions to the vertebrate MBLs and ficolins [11, 27, 41]. Furthermore, although the serine proteases C1r, C1s, and MASPs have not been found in protostomes, MASP-related molecules (MreM) are present in invertebrates [41].

Besides complement activation, FreD-C and C-lectin proteins are also involved in a large variety of immune processes, independent from the complement system. FreD-Cs are a family of proteins characterized by the presence of a fibrinogen domain (FBG) [45]. In vertebrates, besides ficolins, there is a wide variety of FreD-C proteins (e.g., tenascins, angiopoietins), which also have immune functions. In invertebrates, FreD-Cs have been observed to play a role in bacteria agglutination [46]. Moreover, FreD-Cs are also involved in neuronal development and allorecognition [45]. C-lectins are characterized by having at least a C-lectin domain, although other domains can also be present [47]. These proteins are very abundant in invertebrates, and they are involved in a broad variety of immune functions (e.g., agglutination, opsonization, phagocytosis, encapsulation) [48, 49].

The Toll pathway, the Imd pathway, the complement system, and lectins have been well studied in *Drosophila* and vertebrates. However, although some studies have been conducted in mollusks and brachiopods [11, 27, 41], studies on these pathways and systems in other spiralian groups are scarce (Fig. 1D). In nemerteans, our previous study revealed the presence of 6 TLRs in the transcriptome of *Lineus ruber* [13]*.* In this study, we aim to relate the common innate immunity pathways to the immune response in the nemertean *Lineus ruber*. Therefore, we performed a survey in the *Lineus ruber* transcriptome to detect the components belonging to the Toll and the Imd pathways, the complement system, and lectins, and we confirmed the presence of these pathways and systems. Moreover, we studied the expression of the members of these pathways and lectins by in situ hybridization, showing that they are expressed in various organs and tissues. Finally, we performed immune challenge experiments in *Lineus ruber* to study the changes in the expression of some of these genes in response to bacterial infection, revealing that all the genes studied, except for *imd*, seem to be involved in immunity against gram-negative bacteria.

## Results

### Presence of orthologs of the Toll and the Imd pathways, the complement system, and lectins components in *Lineus ruber*

We performed transcriptomic surveys to identify *Lineus ruber* components of the Toll and the Imd pathways, the complement system, and lectins. All protein sequences retrieved from the surveys are available in Additional file 2.

#### The Toll pathway is present in *Lineus ruber*

**Fig. 2:**
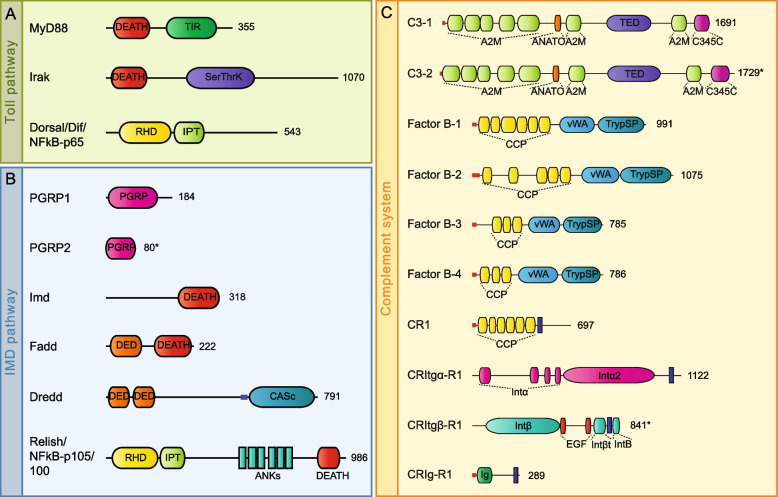
Domain architecture analyses of putative proteins belonging to the Toll and Imd pathways and the complement system in *Lineus ruber*. **A** Proteins belonging to the Toll pathway. **B** The Imd pathway and **C** the complement system. An example of each complement receptor type (CR, CRItgα, CRItgβ, and CRIg) is shown. For other complement receptor proteins identified in this study, see Additional file 5: Fig. S2. Numbers indicate the length of the protein in amino acids. Asterisks after the amino acid number indicate partial proteins. A2M, α2-macroglobulin family domain; ANATO, anaphylatoxin homologous domain; ANK, ankyrin domain; CASc, caspase domain; CCP, complement control protein; DED, death effector domain; EGF, epidermal growth factor domain; Ig, immunoglobulin domain; Intα, integrin-α domain; Intβ, integrin-β domain; Intβt, Integrin-β tail domain; IPT, Ig-like, plexin, transcription factors domain; PGRP, peptidoglycan recognition protein domain; RHD, Rel homology domain; SerThrK, serine/threonine protein kinase domain; TED, thioester domain; TIR, Toll-interleukin 1 receptor domain; TrypSP, trypsin-like serine protease domain; vWA, von Willebrand factor type A domain. Small blue and red bars represent coiled coils and signal peptides, respectively. Blue rectangles represent the transmembrane domains

First, we performed a transcriptomic survey to detect the components of the Toll pathway in the nemertean *Lineus ruber* transcriptome. We identified a *myD88*, an *irak* gene, and the transcription factor *dorsal/diff/NFkB-p65*. Domain architecture (Fig. 2), BLAST (Additional file 3: Table S2), and phylogenetic analyses (Additional file 4: Fig. S1) confirm the identity of these proteins. The presence of an ortholog of *spätzle*, however, was not detected.

*Lineus ruber* proteins belonging to the Toll pathway contain the characteristic domains of these proteins (Fig. 2A), which are also found in ortholog proteins of other species. The *Lineus ruber* MyD88 ortholog contains the TIR and DEATH domains characteristic of MyD88 proteins [50], whereas a DEATH and a serine/threonine protein kinase domain, typical features of the Pelle/Irak1 and Irak4 proteins [51, 52], were identified in the *Lineus ruber* Irak ortholog. Furthermore, two proteins belonging to the NFκB family were also identified, with our phylogenetic analysis of NFκB proteins showing that one of them is an ortholog to the *Drosophila* Dorsal and Dif proteins and the vertebrate NFκB-p65 (Additional file 4: Fig. S1). This protein contains two Relish domains (RHD and IPT domains), which are also found in its orthologs [53]. The second protein was identified as the Relish/NFκB-p105/100 ortholog, the transcription factor of the Imd pathway (see below).

#### Key components of the IMD-like pathway are present in *Lineus ruber*

Next, we performed a survey in the *Lineus ruber* transcriptome to identify the potential components of the IMD pathway. Our results show the presence of two PGRPs and one *imd*, *fadd*, *dredd*, and *relish/NFκB-p105/100* genes.

Our survey identified two PGRPs genes, named *PGRP-1* and *PGRP-2*. Our analyses show that *PGRP-1* encodes a short protein that contains one PGRP domain but lacks the transmembrane domains, signal peptide, or RHIM motifs (Fig. 2B), elements that are present in *Drosophila* PGRP receptors involved in the Imd pathway [24]. Furthermore, PGRP-1 BLAST best hit is the short PGRP-S2 of the mollusk *Hyriopsis cumingii* (Additional file 3: Table S2). Thus, PGRP-1 is a short PGRP, which is probably not involved in Imd pathway activation. Since from our survey we could only obtain a partial sequence for *PGRP-2*, it was not possible to determine if a transmembrane domain and a RHIM motif are present in the PGRP-2 protein. Furthermore, a survey for RHIM motifs in the *Lineus ruber* transcriptome did not detect any sequence encoding this motif. However, the lack of long PGRPs in the transcriptome only shows that these genes are not expressed in that specific stage. Therefore, as no genome of *Lineus ruber* is available, we performed surveys for PGRP domains and RHIM motifs in the genome of the nemertean *Notospermus geniculatus* [54]. Our results show the presence of 8 genes encoding for PGRP domain-containing proteins (Additional file 6: Fig. S3A), but genes encoding for proteins containing RHIM were not detected. All *Notospermus geniculatus* PGRP proteins are shorter than 350 amino acids, with the exception of PGRP5, which is 517 amino acids long. According to Dziarski and Gupta [55], short PGRPs have an approximate length of 200 amino acids, while long PGRPs are at least double in length. Domain architecture analyses show that *Notospermus geniculatus* PGRP5 has a signal peptide and a C-terminal PGRP domain, but no transmembrane domains. Therefore, we suggest that this protein is an extracellular protein which is not involved in Imd pathway activation. Furthermore, we performed a phylogenetic analysis of PGRP proteins, including the *Lineus ruber*, *Notospermus geniculatus*, and *Drosophila melanogaster* PGRPs (Additional file 6: Fig. S3B). This analysis shows that all nemertean PGRPs cluster together forming a sister clade to the *Drosophila melanogaster* short PGRPs and PGRP-LB, proteins that are not involved in Imd pathway activation. Therefore, our results suggest that nemertean PGRPs are probably not involved in Imd pathway activation.

Furthermore, we identified an *imd* gene in the *Lineus ruber* transcriptome. The *Lineus ruber* Imd protein contains the characteristic DEATH domain of Imd proteins (Fig. 2B). Moreover, our survey also retrieved the presence of a *fadd* and a *dredd* genes. Our analyses show that Fadd and Dredd proteins contain Death effector domains, and, in the case of Dredd, one Caspase domain was additionally found, as in the *Drosophila* ortholog [56] (Fig. 2B). Finally, as mentioned above, two NFκB genes are present in the *Lineus ruber* transcriptome. Our analyses identified one of these NFκB proteins as the ortholog of Relish/NFκB-p105/100 (Fig. 2B; Additional file 4: Fig. S1). This protein contains two Relish homology domains (RHD and IPT domains); six ANK repeats—domains that are also present in the *Drosophila* and vertebrate orthologs [53, 57]; and a DEATH domain, which has also been observed in the Relish protein of other arthropods [58, 59].

#### The complement system is present in *Lineus ruber*

Our *Lineus ruber* transcriptomic survey revealed 2 *C3* genes, 4 *complement factor B* (*Cfb*) genes, and up to 26 putative genes encoding for complement receptors (CR) (Fig. 2C, Additional file 5: Fig. S2). Genes encoding for ortholog proteins to the serine proteases C1s/C1r/MASP/MReM and the vertebrate membrane attack complex proteins (C6-9) were not identified. For the latter, this result was expected, as these proteins have only been found in deuterostomes.

Our analyses show the presence of 2 *C3* genes in the transcriptome of *Lineus ruber* (named here *C3-1* and *C3-2*) (Fig. 2C, Additional file 3: Table. S2, Additional file 4: Fig. S1). Domain architecture analyses show that these proteins contain α2-macroglobulin domains, an anaphylatoxin domain, a thioester region, and a C345C C-terminal domain (Fig. 2C). Furthermore, we also unraveled the presence of four *cfb* genes encoding for 4 factor B proteins (factor B-1 to factor B-4) (Fig. 2C, Additional file 3: Table. S2, Additional file 4: Fig. S1). Domain architecture analyses show that these four proteins are composed of complement control protein domains (CCP), a von Willebrand factor (vWF) type A domain, and a trypsin-like serine protease domain (TrypSP) (Fig. 2C). However, we could not identify *Cfc* genes in the *Lineus ruber* transcriptome. Additionally, we identified up to 26 putative genes encoding for complement receptors (CR) with similar domain composition than the vertebrate complement receptors (Fig. 2C, Additional file 5: Fig. S2). Similarly to vertebrate complement receptors CR1 and CR2 [60], 6 proteins were found containing multiple CCP repeats and a transmembrane domain (CR1 to CR6) (Fig. 2C, Additional file 5: Fig. S2). Furthermore, in vertebrates, integrin-α (CD11b) and β (CD18) proteins assemble to form the complement receptors CR3 and CR4 [61]. Here, we also identified 4 transmembrane proteins containing integrin-α or β domains (CR-Itgα1 and 2; and CR-Itgα1 and 2β) (Fig. 2C, Additional file 5: Fig. S2). However, whether these proteins heterodimerize to constitute complement receptors in *Lineus ruber* is not assessed in this study. Moreover, the vertebrate CRIg are constituted by one or more immunoglobulin domains and a transmembrane domain [62]. Here, we show the presence of 16 genes encoding for proteins with similar domain composition (Fig. 2C, Additional file 5: Fig. S2).

#### Putative activators of the complement system

In order to investigate the possible pathways by which the complement system could be activated in *Lineus ruber*, we performed a transcriptome survey to identify the genes encoding for proteins containing FBG, C-lectin, or C1q domains, since these domains are present in vertebrate proteins involved in complement system activation [33, 34].

Our survey reveals 14 genes encoding for proteins containing a FBG domain (FreD-C1 to FreD-C14). While all these proteins have a FBG domain, only FreD-C1 contains a CCP domain (Additional file 7: Fig. S4). Moreover, although the remaining proteins do not have any other domains, FreD-C3, FreD-C4, FreD-C7, and FreD-C11 contain coiled-coil motifs and, therefore, belong to the FreDC2 subfamily [41]. Proteins belonging to this subfamily have been suggested to form multimeric proteins, similarly to the vertebrate ficolins, that could activate the complement system [41]. Our results also show the presence of 39 C-lectin genes in the transcriptome of *Lineus ruber* (*c-lectin1* to *c-lectin39*). These genes encode for proteins with a large variability of domain composition (e.g., leucine-rich repeat domains, von Willebrand factor type-A domain, complement control protein modules) (Additional file 7: Fig. S4), being some of these domains also found in vertebrate C-lectins [47]. Additionally, although some C-lectin proteins constituted only by a sole C-lectin domain were also identified, no proteins containing a collagen domain or a coiled-coil region together with a C-lectin domain were found. This suggests that C-lectin proteins would not be involved in complement activation in *Lineus ruber*. Furthermore, we identified three C1q genes (*C1q-1* to *C1q-3*) in the *Lineus ruber* transcriptome. These genes encode for proteins formed by collagen and C1q domains, and therefore, they are C1qL proteins that could activate the complement system (Additional file 7: Fig. S4).

Together, the findings from our transcriptome surveys in *Lineus ruber* confirm the existence of the Toll and Imd pathways, the complement system, and lectins in this organism. The Toll pathway proteins MyD88, Irak, and Dorsal/Dif/NFκB-p65 were identified, as well as the Imd pathway components Imd, Fadd, Dredd, and Relish/NFκB-p105/100. However, no putative receptors for this pathway were identified. Our results also show the presence of the necessary components to constitute a functional complement system, since C3, factor B, and complement receptors were identified. Additionally, the presence of coiled-coil motifs in FreD-C proteins and collagen domains in C1q proteins suggest a possible involvement of these proteins in complement system activation.

### Genes with putative immune functions are expressed in a variety of tissues in *Lineus ruber*

To study the expression of the aforementioned genes, whole-mount in situ hybridization (WMISH) was performed in 40 and 60 days *Lineus ruber* juveniles (Fig. 4). As with all nemerteans, *Lineus ruber* have an eversible proboscis used to catch prey [64]. *Lineus ruber*’s nervous system is formed by a brain and two lateral and dorsal nerve cords, as well as cephalic nerves that emerge from the brain and extend to the anterior area of the head, innervating the frontal sensory organ and eyes [64–67]. Furthermore, this species possesses a closed circulatory system that consists of two lateral and dorsal blood vessels that run parallel to the lateral and the dorsal nerve cords [64]. By the anterior part, these vessels are connected near the brain and form a cephalic vascular loop surrounding the proboscis. The mouth is in the anterior area of the trunk, opening to the gut, which is extended to the posterior part of the animal. Previous publications and additional experiments in this study (Additional file 8: Fig. S5) show that these organs and systems are already present in *Lineus ruber* juveniles.

**Fig. 3:**
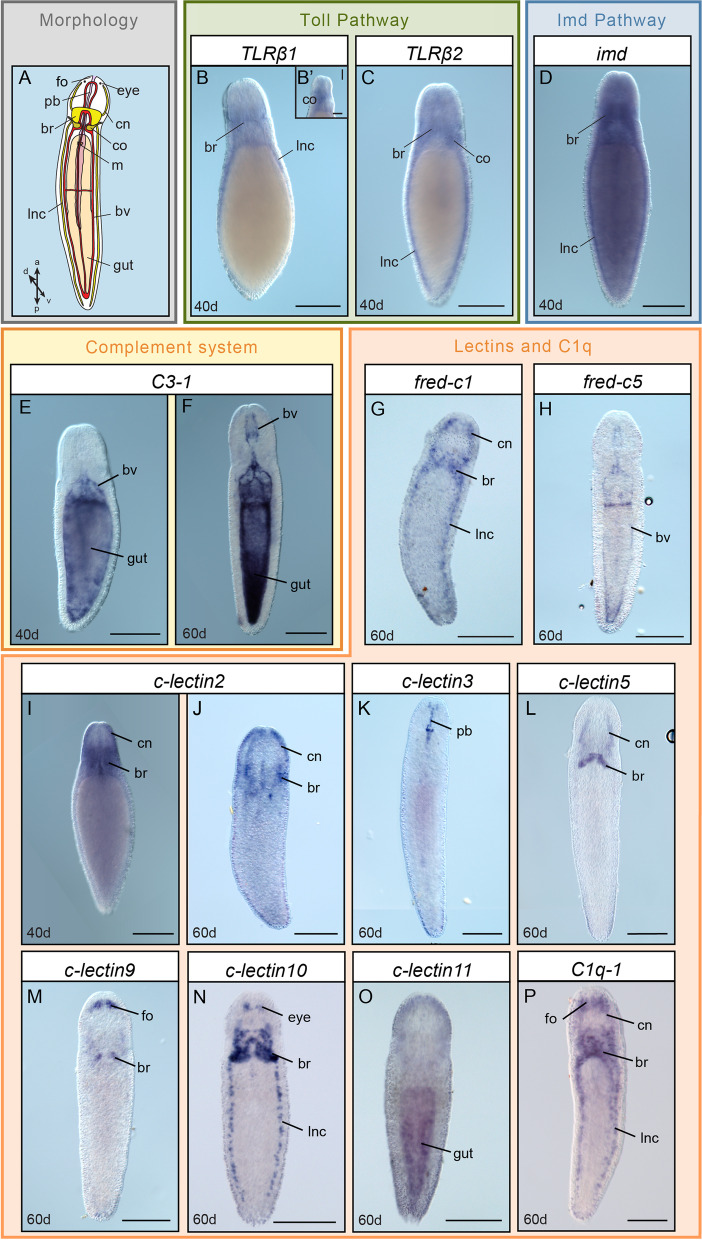
Expression of immune genes in *Lineus ruber*. **A** Schematic representation of *Lineus ruber* anatomy. **B**–**P** Whole mount in situ hybridization (WMISH) of **B**, **C** genes belonging to the Toll pathway and **D** to the Imd pathway and **E**, **F** of the complement system and **G**–**P** lectins and the C1q family. The name of each gene is indicated above each panel. Besides **B’**, all panels show dorso-ventral (d,v) views, being anterior (a) orientation to the top. **B’** Lateral (l) orientation. Scale bars indicate 250 μm, except for the scale bar in **B’**, which indicates 100 μm. br, brain; bv, blood vessel; cn, cephalic nerve; fo, frontal sensory organ; g, gut; l: lateral view; lnc, lateral nerve cord; m, mouth; pb, proboscis. Scheme drawn after [64]

The results of our whole mount in situ hybridization reveal that both *TLRβ1* and *TLRβ2* are expressed in the lateral nerve cords as well as in the brain and cephalic organs in 40-day juveniles (Fig. 3A, B, B’)*.* Similarly, *imd* is also expressed in the brain and the lateral nerve cords (Fig. 3D). Furthermore, the complement *C3-1* gene has been found to be expressed in the gut and the blood both in 40- and 60-day juveniles (Fig. 3E, F). Although at 60 days of development, the expression of *C3-1* is strong in the cephalic vascular loop, at 40 days, it is very faint in this region. In 60-day juveniles, *fred-c1* is expressed in the brain, the ventrolateral nerve cords, and the cephalic nerves (Fig. 3G), whereas *fred-c5* is expressed in the blood (Fig. 3H). The expression of *c-lectin2* was detected in the brain and the cephalic nerves at both stages analyzed (Fig. 3I, J). *c-lectin3* is expressed in the proboscis area of the head (Fig. 3K). *c-lectin5*, *c-lectin9*, and *c-lectin10* are expressed in the nervous system, with *c-lectin5* expressed in areas of the brain and in the cephalic nerves (Fig. 3L); *c-lectin9* in a pair of brain lobes and in the frontal organ (Fig. 3M); and *c-lectin10* in the brain, in the two ventrolateral nerve cords, and in the eyes (Fig. 3N). *c-lectin11* expression was found to be expressed in the gut (Fig. 3O) and *C1q-1* in the brain, the ventrolateral nerve cords, the cephalic nerves, and the frontal sensory organ (Fig. 3P).

Moreover, we also performed in situ hybridization for genes encoding other components of the Toll pathway (*TLRα1*, *TLRα2*, *TLRα3*, *TLRα4*, *myd88*, and *dorsal/dif/NFκB-p65*), the Imd-like pathway (*relish/NFκB-p105/100*), other FreD-Cs (*fred-c2*, *fred-c3*, *fred-c4*, *fred-c6*, and *fred-c7*), C-lectins (*c-lectin1*, *c-lectin4*, *c-lectin5*, *c-lectin6*, and *c-lectin7*), and the C1q family member *C1q-2*, but no expression was obtained. This could be explained by the absence or very low expression levels, non-detectable by WMISH, of these genes in healthy juvenile animals.

### The degree of expression of immune-related genes is altered in infected *Lineus ruber*

To understand whether the candidate genes were involved in immunity against gram-negative bacteria, we exposed healthy adult *Lineus ruber* specimens to *Vibrio diazotrophicus* for 3 h, 6 h, 12 h, and 24 h. The expression levels of the *TLRα3*, *TLRα4*, *TLRβ1*, *TLRβ2*, *imd*, *fred-c5*, *C3-1*, and *c-lectin2* genes were evaluated at those time points performing quantitative real-time PCR (qPCR) and compared between control and infected animals.

**Fig. 4:**
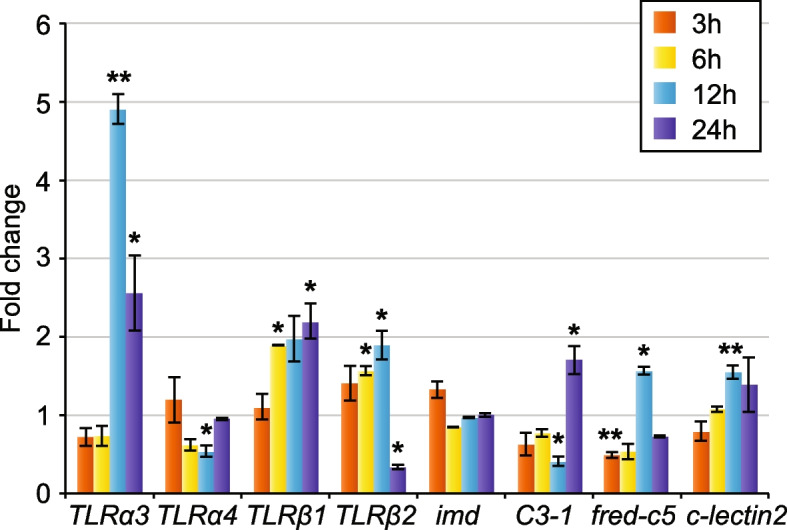
Relative expression of immune genes in response to infection to *Vibrio diazotrophicus*. Expression of immune genes in the different time points was normalized to the gene expression levels in the control animals. The fold change was calculated using the 2^−ΔΔCT^ method [63], standardizing to 1 the expression level for control animals. Asterisks indicate significant differences in the gene expression between control and infected animals, being evaluated by performing statistical ANOVA tests (*p-value < 0.05; **p-value < 0.01). Bars indicate the standard error between infected biological replicates

Our results show that the expression of most genes was not significantly altered at 3 h of infection, except for *fred-c5* (Fig. 4). *TLRα3* expression levels were upregulated in infected animals at 12 h, remaining increased at 24 h of infection. In contrast, *TLRα4* expression was downregulated at 12 h of infection and never upregulated at the time points of study. *TLRβ1* expression levels were increased at 6 h and remained such at 12 h and 24 h of infection. Interestingly, *TLRβ2* was upregulated at 6 h and 12 h of infection, but its expression decreased by 24 h of infection. *imd* expression did not vary at any of the studied time points. The complement factor *C3-1* was downregulated at 12 h of infection, but its expression levels were increased by 24 h of infection. *fred-c5* expression was downregulated already at 3 h of infection; however, its expression increased at 12 h and reached similar expression levels to control animals at 24 h of infection. *c-lectin2* expression was upregulated at 12 h in infected animals, while its expression dropped to similar expression levels to control animals at 24 h of infection.

Thus, our results show that the gram-negative bacteria *Vibrio diazotrophicus* triggers an immune response in the nemertean *Lineus ruber*, in which the Toll receptors, the complement system, and the lectins *fred-c5* and *c-lectin2* are involved. However, although the Imd pathway is involved in defense against gram-negative bacteria in arthropods [21–23], the *imd* gene expression remained unaltered in all the studied time points.

## Discussion

### The Toll pathway is involved in the gram-negative immune response in *Lineus ruber*

**Fig. 5:**
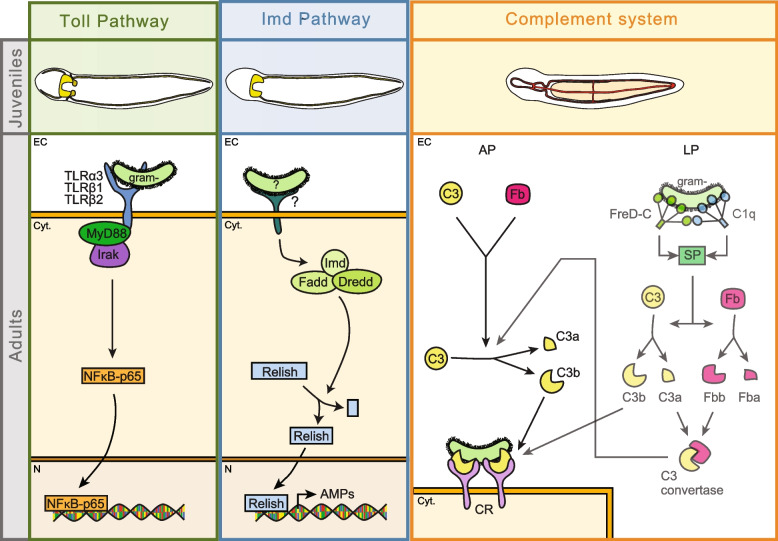
The Toll pathway, Imd pathway, and complement system in *Lineus ruber.* “?” indicates uncertainty about the identity of the agent activating the pathway or the receptor involved in it. Semi-transparent schemes indicate the uncertainty in the existence of this pathway. AP, alternative pathway; CR, complement receptor; Cyt., cytoplasm; EC, extracellular space; Fb, factor B; gram-, gram-negative bacteria; LP, lectin pathway; N, nucleus; SP, serine protease

The Toll pathway is a pathway involved in immunity that is present across many metazoan lineages [4, 7–9]. In a previous study, we unraveled the presence of 6 TLRs in the nemertean *Lineus ruber* [13]. In this study, we identified the presence of the MyD88 adaptor, an Irak protein and a Dorsal/Diff/NF-κB-p65 protein in this nemertean species, but no Spätzle protein ortholog was identified (Fig. 2A). Therefore, we suggest that TLRs in *Lineus ruber* are probably activated directly by the pathogen, similar to other spiralians and deuterostomes [8]. Considering our results under the scope of the existing knowledge on the Toll pathway in other animals, we suggest that, once the *Lineus ruber* TLRs are activated, a signaling cascade in which MyD88 and an Irak protein are involved triggers the entrance of the *Lineus ruber* Dorsal/Diff/NF-κB-p65 into the nucleus (Fig. 5). Moreover, our results show that *TLRβ1* and *TLRβ2* are expressed in the nervous system in *Lineus ruber* juveniles (Figs. 3 and 5). Therefore, at this stage, these receptors could be involved in immunity and/or in nervous system development, as TLRs have been shown to also be involved in the development of the nervous system in other metazoans, including cnidarians, arthropods, and vertebrates [19, 20, 68–70]. Additionally, upon exposure to the gram-negative bacteria *Vibrio diazotrophicus*, *TLRα3*, *TLRβ1*, and *TLRβ2* are upregulated, whereas *TLRα4* expression did not vary or was downregulated (Fig. 4). These findings show that at least three TLRs in *Lineus ruber* are involved in the gram-negative bacterial response. Although the Toll pathway is not involved in *Drosophila*’s defense against gram-negative bacteria, upregulation of TLRs in *Lineus ruber* against gram-negative infection is in agreement with findings in other invertebrate species, such as other arthropods and mollusks [14, 15]. Furthermore, *TLRα4* could be either involved in other stages of infection against gram-negative bacteria or involved in the detection of other pathogens (e.g., gram-positive bacteria, fungi, virus) or not be involved in immunity.

### The Imd-like pathway is present in *Lineus ruber*, but it seems to not be involved in immunity against gram-negative bacteria

The Imd pathway has been shown to be a pivotal pathway in the defense against gram-negative bacteria in arthropods [21–23]. However, no orthologs of the Imd protein, the key component of this pathway, have been found in spiralians and vertebrates [7, 11, 14, 25, 27]. In this study, we surveyed for components of this pathway in the transcriptome of the nemertean *Lineus ruber*, identifying for the first time an *imd* ortholog in spiralians (Fig. 2B). Besides *imd*, we also found the downstream components *fadd*, *dredd*, and *relish/NFκB-p105/100*. Except for the Imd protein, orthologs of proteins belonging to the arthropod Imd pathway have also been identified in mollusks, brachiopods, and vertebrates [7, 14, 25, 27]. Among these proteins, transmembrane PGRPs compatible with Imd pathway activation are present both in brachiopods and mollusks [11, 27]. However, although our analysis identified 2 PGRPs in the *Lineus ruber* transcriptome and 8 in the *Notospermus geniculatus* genome (Additional file 6: Fig. S3), we did not find evidence for them to be the receptors of this pathway. Therefore, although our findings might indicate the existence of this pathway in nemerteans, this pathway would be activated by other receptors than PGRPs (Fig. 5B). Furthermore, our results show that *imd* is expressed in the nervous system in juveniles (Figs. 3 and 5). However, although this gene is upregulated after gram-negative bacterial exposure in arthropods [7, 22, 23], exposure of adult *Lineus ruber* to the gram-negative bacteria *Vibrio diazotrophicus* did not result in differences in the expression of this gene (Fig. 4). These results indicate that the Imd pathway is probably not involved in gram-negative response in *Lineus ruber* or it is involved in other time points of infection not tested here. Furthermore, its involvement in the immune response towards other types of pathogens cannot be excluded.

### The complement system is involved in *Lineus ruber* immunity against gram-negative bacteria

Previous studies show the presence of a complement system formed by C3 and factor B genes across the metazoan tree, including cnidarian [71], spiralian [11, 27, 38, 39], ecdyzoan [72, 73], and deuterostome species [74, 75] (Fig. 1D). Our study shows that two C3 genes and four Cfb genes are present in the transcriptome of the nemertean *Lineus ruber* (Fig. 2C). Furthermore, up to 26 putative genes encoding for complement receptors proteins with similar domain architectures to the human complement receptors [60–62] were also detected in our *Lineus ruber* transcriptomic survey (Fig. 2C; Additional file 5: Fig. S2). Since C3, factor B, and complement receptors constitute the core components of the alternative pathway, the presence of these proteins in *Lineus ruber* suggests the presence of this complement pathway in this species (Fig. 5C).

Although the lectin pathway is thought to have been emerged in early chordate evolution [44], recent studies suggest the presence of this pathway in spiralians [27, 41]. Here, we show that multiple FreD-C and C-lectin proteins are present in *Lineus ruber* (Additional file 7: Fig. S4). Although no collagen domains have been found within these proteins, a domain that is always present in vertebrate FreD-C and C-lectins activating this pathway [76, 77], four proteins with coiled-coil regions and an FBG domain were detected (FreD-C3, FreD-C4, FreD-C7, and FreD-C11). Previous studies have shown that FreD-C and C-lectin proteins containing coiled-coil motifs are multimeric proteins that are also present in other spiralians and suggested their involvement in complement activation [11, 27, 41, 78]. Therefore, it is plausible that *Lineus ruber* FreD-Cs could activate the complement system via the lectin pathway (Fig. 5C).

Furthermore, the classical pathway to activate the complement system is also considered to be a vertebrate innovation, since antibodies of adaptive immunity are often involved in this pathway [44, 79]. However, as activation of the complement system by C1q is not always antibody-dependent [28, 80] and C1q proteins containing collagen domains are widespread in metazoan species [11, 38, 41], it has been suggested that C1q proteins could activate the complement system in invertebrates in an antibody-independent way [27, 41]. Here, we found 3 genes encoding for C1q proteins containing a collagen domain in the *Lineus ruber* transcriptome (Additional file 7: Fig. S4), suggesting that these proteins could be putative activators of the complement system. However, although the complement system could likely be activated in *Lineus ruber* either by FreD-C and/or C1q proteins, the mechanism to circumvent the lack of the serine proteases MASP, C1r, and C1s and cleave of C3 and factor B to form the C3 convertase has yet to be elucidated. It has been suggested that MreM could perform this function in other spiralians [41]. However, we did not find MreM genes in the transcriptome of *Lineus ruber*.

Furthermore, our results show that the complement gene *C3-1* is expressed in the blood and the gut in *Lineus ruber* juveniles (Figs. 3 and 5). Additionally, we show that this gene is upregulated in adult *Lineus ruber* after exposure to *Vibrio diazotrophicus* (Fig. 4), suggesting that *Lineus ruber* complement could be activated in response to gram-negative bacterial infection. This is in concordance with previous studies showing the upregulation of complement components after exposure to gram-negative bacteria in other invertebrates, such as cnidarian, mollusks, and invertebrate deuterostomes [38, 81–84]. Additionally, the upregulation of complement system components in invertebrate deuterostomes and mollusks has also been observed to occur after exposure to gram-positive bacteria [82, 84]. However, in this study, activation of the complement system by other pathogens (e.g., gram-positive bacteria, fungi) was not assessed and, therefore, cannot be excluded.

### FreD-C and C-lectin proteins, likely not part of the complement system activation, could also be involved in immunity in *Lineus ruber*

*FreD-C and C-type lectins are proteins with high structural and functional diversity* [47, 48, 85]. In our study, besides the presence of FreD-Cs putatively involved in *Lineus ruber* complement activation (see above), we show the presence of 10 FreD-Cs and 39 C-lectins with domain architectures not suitable for this function (Additional file 7: Fig. S4). Therefore, these proteins must have other functions than complement activation. Here, we show that both *fred-c5* and *c-lectin2*, expressed in the blood and the head nervous system, respectively (Fig. 3C), are upregulated after gram-negative bacterial exposure (Fig. 4), and therefore, they are involved in immunity against this type of bacteria. Other FreD-Cs and C-lectins are expressed in various tissues (e.g., gut, anterior proboscis, and nervous system) (Fig. 3), suggesting that they could be involved in immunity in those tissues. Expression of immune genes in various systems, including the blood, the gut, and the nervous system, in *Lineus ruber* is not surprising, since immunity in animals is not restricted to blood, hemolymph, or the lymphatic tissues, but immune genes are also expressed in many other tissues that need defense mechanisms against pathogens [86–88]. This is especially important for tissues such as the gut, which is continuously exposed to microorganisms [86, 89, 90]. Moreover, genes involved in immunity have also been shown to be expressed in the nervous system and sensory structures in other organisms [91–93].

## Conclusions

In this study, we identified key components of the Toll and the Imd pathways and the complement system in the nemertean *Lineus ruber*. The presence of the complement system C3, factor B, and complement receptor proteins indicates that complement could be activated by the alternative pathway, whereas the presence of C1q and FreD-C proteins with characteristics resembling those ones that activate the complement system in vertebrates suggests that this system could also be activated by the lectin pathway. Moreover, the upregulation of genes belonging to the Toll pathway and the complement system after exposure to *Vibrio diazotrophicus* suggests that these pathways and systems could be involved in immunity against gram-negative bacteria in *Lineus ruber*. We demonstrate the presence of the Imd pathway in spiralians, identifying the Imd protein in *Lineus ruber*. However, expression levels of *imd* were not affected during *Vibrio diazotrophicus* infection, suggesting that this pathway might not be involved in defense against gram-negative bacteria in *Lineus ruber*. Additionally, lectins, probably not involved in complement system activation, could be involved in immunity (e.g., *fred-c5*, *c-lectin2*). Overall, our results demonstrate the presence of immune pathways involved in defense against gram-negative bacteria in *Lineus ruber*. However, further research is necessary in order to elucidate the role of these pathways in response to other pathogens (e.g., gram-positive bacteria, fungi) and other putative roles in the organism.

## Methods

### Animals and bacteria

Adult *Lineus ruber *(Müller, 1774) [94] were collected during winter on a rocky beach in Bergen, Norway (coordinates: 60° 15′ 06.6″ N 5° 19′ 15.4″ E). The animals are kept in the animal facility in sea water tanks at 10–12 °C and salinity 33 with a constant air supply. Once per week, they were fed with mussels and the water was changed. During March to April, when oviposition occurred, cocoons were collected and cultured in the same salinity and temperature conditions than the adults, but they were never fed. Juveniles were fixed at 60 days after oviposition (dao) for whole-mount in situ hybridization. First, animals were relaxed in 7.4% MgCl_2_ and then fixed in 4% formaldehyde during 1 h at room temperature. The fixative was washed repeatedly with phosphate buffer saline 0.1% Tween-20 (PTw), and specimens were stored at − 20 °C in 100% methanol. For histological methods, animals were stored in 100% ethanol instead.

The gram-negative bacteria *Vibrio diazotrophicus* were purchased from ATCC (catalog number: 33466). The bacteria were resuspended and cultured in Difco™ Marine Broth (Fisher Scientific) at 26 °C overnight.

### Bioinformatic survey of immune genes in *Lineus ruber*

Alignments for conserved domains of the proteins of interest were downloaded from Pfam database [95]. When alignments were not available for our protein of interest in pfam, orthologs of our proteins of interest were collected in the NCBI database (www.ncbi.nlm.nih.gov), and alignments were built with the MAFFT software version 7 [96]. Hmmer profiles were built from alignments of the protein of interest using the HMMER software v3.2.1 (www.hmmer.org) and blasted against the *Lineus ruber* transcriptome that is composed out of mixed embryonic stages and adults and was assembled with Trinity that also detects splice variants (reference Martin-Duran et al., Gasiorowski et al., and your last paper) (SRA PRJNA881742) [97] the *Notospermus geniculatus* genome [54]. For those proteins for which alignments were not available for their conserved domains, the full sequence of vertebrate and *Drosophila* orthologs were blasted into the *Lineus ruber* transcriptome. The sequences obtained from these surveys were validated by BLAST [98] (www.blast.ncbi.nlm.nih.gov). Domain architecture organization was analyzed with SMART [99, 100] (http://smart.embl.de), hmmer [101] (http://hmmer.org), and NCBI Conserved Domains [102] (https://www.ncbi.nlm.nih.gov/Structure/cdd/wrpsb.cgi).

### Phylogenetic analyses

Amino acid sequences from *Lineus ruber* were obtained from the bioinformatic survey of immune genes. Sequences from other species were obtained from the NCBI database (www.ncbi.nlm.nih.gov). Sequences were aligned using the MAFFT software version 7 [96], using the L-INS-I algorithm. The alignment was trimmed with the TrimAl software version 1.2 [103]. Phylogenetic analyses were performed using the maximum likelihood IQ-TREE software [104] in the CIPRES Science Gateway V.3.3 [105] (http://www.phylo.org). For the phylogenetic analysis of DEATH domain-containing proteins, LG+F+I+G4 was selected as the best-fit model according to Bayesian Information Criterion (BIC), whereas for the phylogenetic analysis of Nfκb factors VT+I+G4 was selected as the best-fit model. For both the phylogenetic analysis of proteins belonging to the TEP family and the one for factor B, factor C, and factor L proteins, LG+R4 was chosen as the best-fit model, whereas the LG+G4 model was chosen for the PGRP phylogenetic analysis. Bootstrap values were calculated running 1000 replicates using ultrafast bootstrap.

### Gene cloning and probe synthesis

Specific primers for each gene were designed using the MacVector 10.6.0 software based on sequences obtained from transcriptomic surveys. Fragments of each gene of interest obtained by amplification of cDNA libraries from adult and juvenile stages. The fragments were inserted into pGEM-T Easy vectors (Promega, USA) and transformed into competent *E. coli* cells. Minipreps were prepared using NucleoSpin®Plasmid kit (Macherey-Nagel) and sequenced in the Sequencing facility of the University of Bergen. RNA probes were transcribed using digoxigenin-11-UTP (Roche, USA) with the MEGAscript™ kit (Invitrogen, Thermo Fisher).

### Histology: embedding, sectioning, and hematoxylin-eosin staining

Specimens were embedded in paraffin by the Molecular Imaging Facility (MIC) of the University of Bergen. The embedding consisted of two incubations of 7 min each with Neo-Clear Xylene substitute (Sigma Aldrich) followed by three incubation steps of 20 min each in melted paraffin at 60 °C. On the last step, the specimens were transferred into cassettes with paraffin and moved to RT for the paraffin to solidify. Next, horizontal cross-sections of 7 μm thickness were made using a microtome Leica RM2255. The sections were transferred into poly-I-lysine coated slides (Thermo Scientific™ SuperFrost Plus™) and dried overnight at 37 °C. Next, sections were deparaffinated by immersion into Neo-Clear Xylene substitute (Sigma Aldrich), followed by descending ethanol series (100%, 96%, 70%) and incubation in phosphate buffer saline (PBS). Hematoxylin-eosin (H-E) staining was performed incubating the samples in hematoxylin (Sigma Aldrich) for 5 min and in eosin (Sigma Aldrich) for 30 s. The slides were washed with PBS after both stainings and mounted in 70% glycerol. Samples were imaged with an Axioscope Ax5 (Zeiss, Oberkochen, Germany).

### Whole-mount in situ hybridization (WMISH)

WMISH were performed as described elsewhere [97]. Proteinase K digestion was performed during 15 min. Probes were hybridized at a concentration of 1 ng/μl at 67 °C during approximately 72 h. Anti-digoxigenin-AP antibody (1:5000) was used for probe detection, and in situs were developed using NBT/BCIP. Samples were washed twice in 100% ethanol and hydrated in descending ethanol steps (75%, 50%, and 25%). Next, 3 washes in PBS were performed and they were incubated in 70% glycerol overnight. Samples were mounted in 70% glycerol.

### Imaging

Histological sections and colorimetric in situ hybridization were imaged using an Axiocam HRc camera connected to an Axioscope Ax10 (Zeiss, Oberkochen, Germany).

### Immune-challenge experiments in *Lineus ruber*

Immune-challenge experiments in *Lineus ruber* were designed using a similar approach to other previous studies in which immune-challenge experiments were performed [84, 86, 88, 106–110]. Immune-challenge experiments were performed in *Lineus ruber* adult specimens collected specifically for this experiment. The animals were acclimatized for 2 weeks in the animal facility, in the same conditions as described before, prior to the experiment. Bacterial concentration was assessed by monitoring animals for 48 h at different concentrations (10^6^ bacteria/ml, 10^7^ bacteria/ml, 10^8^ bacteria/ml, and 7.6 × 10^8^ bacteria/ml). The highest concentration was found to be lethal after approximately 3 h of exposure, while animals in the remaining concentrations survived for 48 h. Thus, we selected a concentration of 10^8^ bacteria/ml for the immune challenge experiments. Sixty-four animals were randomly distributed into 8 groups of 8 animals each. Four groups were exposed to *Vibrio diazothropic*us (10^8^ bacteria/ml of sea water), while the other 4 groups were used as controls. Control animals were kept in autoclaved sea water. Prior to infection, both control and immune-challenged animals were injured with a sterile needle to facilitate the penetration of the bacteria in the infected animals. The animals from one control group and one immune-challenged group were frozen in liquid nitrogen and stored individually at − 80 °C.

### RNA extraction, DNA synthesis, qPCR, and data analysis

mRNA extractions were performed individually for each animal using TRI Reagent^TM^ Solution (Thermo Fisher Scientific) and 1-bromo-3-chloropropane (Sigma). cDNA was synthetized using SuperScript™ III First-Strand Synthesis System (Invitrogen), following the manufacturer’s recommendations. Each reaction contained initially 1 μg of RNA. Specific primers for each gene were designed (MacVector 10.6.0 software) (Additional file 9: Table S3) and tested prior to the experiments. TLR gene sequences were obtained from our previous study Orús-Alcalde et al. [13], whereas the remaining sequences were obtained from the transcriptome survey in this study. qPCRs were performed in Roche LightCycler 480 real-time PCR machine. The master mix contained 1 μl of cDNA, 2 μl of primers (10 μM), 7 μl of sterile RNAse free water, and 10 μl of mastermix Roche Diagnostics Lightcycler 480 Sybr Green I M (Fisher Scientific). *Actin* was searched in the transcriptome and used as a reference gene (Additional file 2). For each technical and biological replicate, the gene of interest was normalized with the actin expression levels. Next, each gene of interest was compared between the infected and control animals for each timepoint, to obtain the fold expression using the 2^−ΔΔCT^ method (Additional file 10: Table S4) [63]. Data was analyzed with the Light Cycler 480 SW 1.5.1, Microsoft Excel, and StatPlus:mac LE v7.

### Illustrations

All figure plates were assembled with Adobe Illustrator CS6.

## Supplementary Information

**Additional file 1: Table S1.** References from Fig. 1.

**Additional file 2:.** FASTA file of Sequences retrieved from the *Lineus ruber* transcriptome survey.

**Additional file 3: Table S2.** BLAST hits from the components of the *Lineus ruber* Toll and Imd pathways and C3 and Factor B proteins from the complement system. The ordinal numbers before each hit indicates the position of each hit. All 1rst positions are indicated, but hits for uncharacterized proteins and repeated proteins are omitted.

**Additional file 4: Fig. S1.** Phylogenetic analyses of the putative components of the Toll-, the Imd- pathways and the complement system. A. Maximum likelihood phylogenetic analysis of DEATH domain containing proteins of the Toll and the Imd pathways. **B.** Maximum-likelihood phylogenetic analysis of Nfκb factors in *Lineus ruber*, *Homo sapiens*, *Mus musculus* and *Drosophila melanogaster.* **C.** Maximum-likelihood phylogenetic analysis of proteins belonging to the TEP family. TEP family is constituted by TEP, C3, and α2M proteins. **D**. Maximum-likelihood phylogenetic analysis of Factor B, C2, Factor C and Factor L proteins. For all trees, dots indicate support values ≥ 60 (black dots: 98-100%; blue dots: 90-97%; green dots: 80-89%; orange dots: 70-79%; pink dots: 60-69%). Tip labels indicate the species name abbreviation followed by the gene name. *Lineus ruber* proteins are labeled in bold. Species abbreviation: Af: *Azumapecten farreri;* Am: *Apis mellifera;* Bb: *Branchiostoma belcheri;* Ce: *Caenorhabditis elegans*; Cf: *Chlamis farreri*; Cg: *Crassostrea gigas;* Ci: *Ciona intestinalis*; Dm: *Drosophila melanogaster;* Ha: *Hasarius adansoni;* Hr: *Halocynthia roretzi;* Hs: *Homo sapiens;* Ir: *Ixodes ricinus;* La: *Lingula anatina;* Lg: *Lottia gigantea; Ll: Littorina littorea;* Lr: *Lineus ruber;* Ls: *Lepidonotus squamatus;* Mc: *Mytilus coruscus;* Mm: *Mus musculus;* Ms: *Melanaphis sacchari*; Nve: *Nematostella vectensis;* Nvi: *Nasonia vitripennis*; Ob: *Octopus bimaculoides;* Pt: *Parasteatoda tepidariorum;* Rd: *Ruditapes decussatus;* Sc: *Sinonovacula constricta;* Sd: *Suberites domuncula*; Spa: *Scylla paramosain;* Spu: *Strongylocentrotus purpuratus;* Ss: *Scylla serrata;* Tt: *Tachypleus tridentatus;* Xl: *Xenopus laevis*.

**Additional file 5: Fig. S2.** Complement receptors in *Lineus ruber*. **A.** Domain architecture analyses of *Lineus ruber* proteins with similar domain architecture than vertebrate CR1 and CR2. **B.** Domain architecture analyses of *Lineus ruber* proteins with similar domain architecture than vertebrate CR3 and CR4. **C.** Domain architecture analyses of *Lineus ruber* proteins with similar domain architecture than vertebrate CRIg. Red rectangles indicate signal peptides. Numbers adjacent to each protein indicate the length of the protein in amino acids and asterisks indicate partial proteins.

**Additional file 6: Fig. S3.** PGRP proteins in *Notospermus geniculatus* and *Lineus ruber.* **A.** Domain architecture analyses of PGRPs in *Notospermus geniculatus.* All the PGRPs in *Notospermus geniculatus* contain only one PGRP domain, with the exception of Ngen_PGRP2 and Ngen_PGRP6. White asterisk indicates that transmembrane domains for Ngen_PGRP1-3 were only detected by hmmer online software and not SMART online software. Red rectangles indicate signal peptides. Numbers adjacent to each protein indicate the length of the protein in aminoacids. **B.** Maximum-likelihood phylogenetic analysis of PGRP proteins in *Lineus ruber (Lr)*, *Notospermus geniculatus (Ngen)* and *Drosophila melanogaster (Dm).* Nemertean PGRP group forming an independent clade than *Drosophila melanogaster* PGRPs. *Drosophila melanogaster* sequences group forming two clades: a clade formed exclusively by long PGRPs and a clade formed by all short PGRPs and a non-transmembrane long PGRP (Dm_PGRP-LB). The later clade is the sister clade to the nemertean PGRPs. Numbers next to the tree nodes indicate bootstrap values. Red labels indicate *Drosophila* PGRPs involved in Imd pathway activation; Blue labels indicate *Drosophila* PGRPs involved in Imd pathway regulation.

**Additional file 7: Fig. S4.** FreD-C, lectins and C1q in *Lineus ruber*. **A**. Fibrinogen-related domain containing proteins (FreD-C). **B.** C-type lectins (C-lectins). **C.** C1q proteins. Numbers adjacent to each protein indicate the length of the protein in aminoacids. Asterisks next to the amino acid number indicate partial proteins. Red rectangles indicate signal peptides; blue small rectangles are coiled coils.

**Additional file 8: Fig. S5.** Morphology of *Lineus ruber* juveniles. **A.** Diagram showing the level of the cross-sections on B-D panels. **B-D.** Hematoxilin-Eosin staining of cross-sections at different points across the anterior-posterior axis. Dorsal is to the top. All scale bars indicate 100μm. bl: blood lacunae; br: brain, co: cephalic organs; dbv: dorsal blood vessel; g: gut; lbv: lateral blood vessel; nc: nerve cord; pb: proboscis; rc: rhynchocoelum; vbl: ventral blood lacunae.

**Additional file 9: Table S3.** qPCR primers.

**Additional file 10: Table S4.** qPCR data. The fold expression (infected vs control animals) was calculated for two or three biological replicates using the 2^-ΔΔCT^ method (Livak and Schmittgen, 2001) and the average fold for each gene and timepoint was calculated. ANOVA tests were performed to test if the expression changes were significant (being p-value<0.05 significant, and *p*-value<0.01 very significant).

**Additional file 11.** Accession Numbers from sequences used for the phylogenetic analyses.

## Data Availability

The datasets supporting the conclusions of this article are included within the article and its additional files. Sequences obtained in the genomic/transcriptomic surveys are available in Additional file 2. Accession numbers used for phylogenetic analyses are provided in Additional file 11. NCBI accession numbers of genes for which their expression was studied by WMISH and qPCR are the following: *TLRα3*: ON146341; *TLRα4*: ON146342; *TLRβ1*: ON146343; *TLRβ2*: ON146344; *imd*: ON146345; *C3-1*: ON146346; *freD-c1*: ON146347; *fred-c5*: ON146348; *c-lectin2*: ON146349; *c-lectin3*: ON146350; *c-lectin5*: ON146351; *c-lectin9*: ON146352; *c-lectin10*: ON146353; *c-lectin11*: ON146354; and *c1q1*: ON146355.

## Abbreviations

CCP: Complement control protein domains
*Cfb*: *Complement factor B*
*Cfc*: *Complement factor C*
CR: Complement receptor
FBG: Fibrinogen domain
FreD-Cs: Fibrinogen-related domain-containing proteins
MBLs: Mannose-binding lectins
MreM: MASP-related molecules
PAMPs: Pathogen-associated molecular patterns
PGRP: Peptidoglycan recognition protein receptor
PGRP-L: Long peptidoglycan recognition protein receptor
PRRs: Pathogen recognition receptors
qPCR: Quantitative real-time PCR
TLR: Toll receptor
TrypSP: Trypsin-like serine protease domain
vWF: Von Willebrand factor
WMISH: Whole mount in situ hybridization
