# Supplementary material for: The localization of Toll and Imd pathway and complement system components and their response to Vibrio infection in the nemertean Lineus ruber
Source: BMC Biol. 2023 Jan 12;21:7. doi: 10.1186/s12915-022-01482-1 (PMC9835746; doi:10.1186/s12915-022-01482-1)
Supplement: Supplementary file 3 — Additional file 3: Table S2. BLAST hits from the components of the Lineus ruber Toll and Imd pathways and C3 and Factor B proteins from the complement system. The ordinal numbers before each hit indicates the position of each hit. All 1rst positions are indicated, but hits for uncharacterized proteins and repeated proteins are omitted. [file 12915_2022_1482_MOESM3_ESM.docx]

**Additional file 3: Table S2. BLAST hits from the components of the *Lineus ruber* Toll and Imd pathways and C3 and Factor B proteins from the complement system.** The ordinal numbers before each hit indicates the position of each hit. All 1rst positions are indicated, but hits for uncharacterized proteins and repeated proteins are omitted.

| **Protein** | **Best hits in BLAST** | **E-value** | **Identity %** |
| --- | --- | --- | --- |
| **MyD88** | *1^st^: Myeloid differentiation primary response protein 88b [Cyprinus carpio]* | 5e^-53^ | 38.93% |
| **Irak** | *1^st^: Receptor-like kinase LIP2 isoform X1 [Mizuhopecten yessoensis]*  *3^rd^: Interleukin-1 receptor-associated kinase 4-like [Lingula anatina]* | 1e^-66^  1e^-54^ | 31.58%  37.50% |
| **Dorsal/NFkB-p65** | *1^st^: Nuclear factor-kB [Cyclina sinensis]*  *2^nd^: Rel/NF-kB [Haliotis discus discus]* | 1e^-144^  1e^-137^ | 58.58%  55.01% |
| **PGRP-1** | *1^st^: Peptidoglycan recognition protein S2 [Hyriopsis cumingii]* | 1e^-70^ | 58.43% |
| **PGRP-2** | *1^st^: Peptidoglycan recognition protein 1 [Gopherus evgoodei]* | 4e^-30^ | 59.74% |
| **Fadd** | *1^st^: FAS-associated death domain protein [Vombatus ursinus]* | 5e^-22^ | 30.14% |
| **Imd** | *1^st^: Imd [Scylla paramamosain]* | 2e^-6^ | 31.33% |
| **Dredd** | *1^st^: Caspase-8 [Lingula anatina]* | 2e^-81^ | 52.87% |
| **Relish/Nfkb-p105/100** | *1^st^: Hypothetical protein CAPTEDRAFT_181359 [Capitella teleta]*  *2^nd^: Nuclear factor NF-kappa-B p105 subunit [Mus musculus]* | 6e^-165^  1e^-159^ | 35.18%  37.20% |
| **C3-1** | *1^st:^ Complement C3 [Branchiostoma belcheri]* | 0 | 33.43% |
| **C3-2** | *1^st^ Venom factor [Lingula anatina]*  *Many complement C3 hits* | 0 | 35.36% |
| **Factor B-1** | *1^st^: complement component 2/factor B variant 2 [Tachypleus tridentatus]* | 1e^-117^ | 30.63% |
| **Factor B-2** | *1^st^: complement factor B-like isoform X1 [Centruroides sculpturatus]* | 1e^-111^ | 29.39% |
| **Factor B-3** | *1^st^: complement factor B-like isoform X2 [Centruroides sculpturatus]* | 5e^-73^ | 26.93% |
| **Factor B-4** | *1^st^: complement C2-like [Branchiostoma floridae]* | 1e^-67^ | 27.99% |
