# Supplementary figures and images for: The localization of Toll and Imd pathway and complement system components and their response to Vibrio infection in the nemertean Lineus ruber
Source: BMC Biol. 2023 Jan 12;21:7. doi: 10.1186/s12915-022-01482-1 (PMC9835746; doi:10.1186/s12915-022-01482-1)

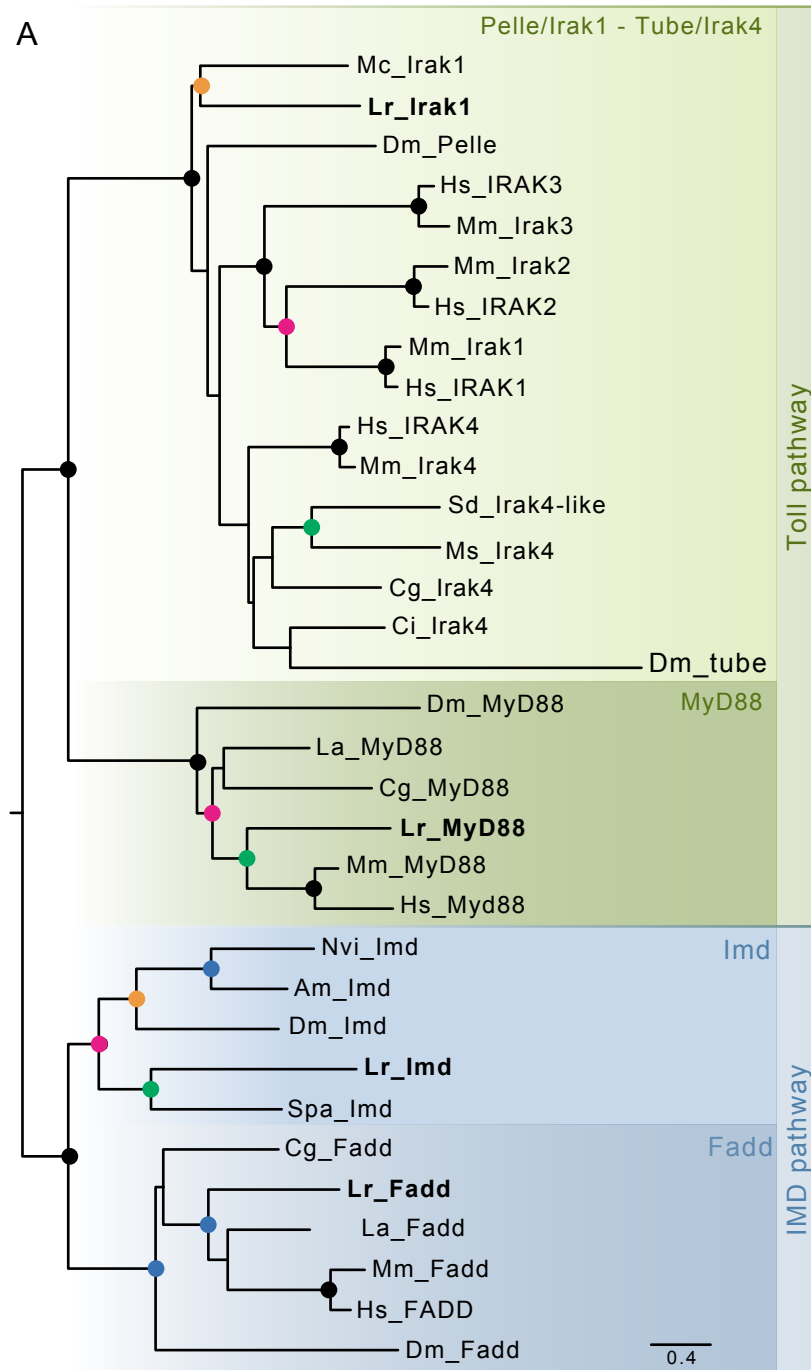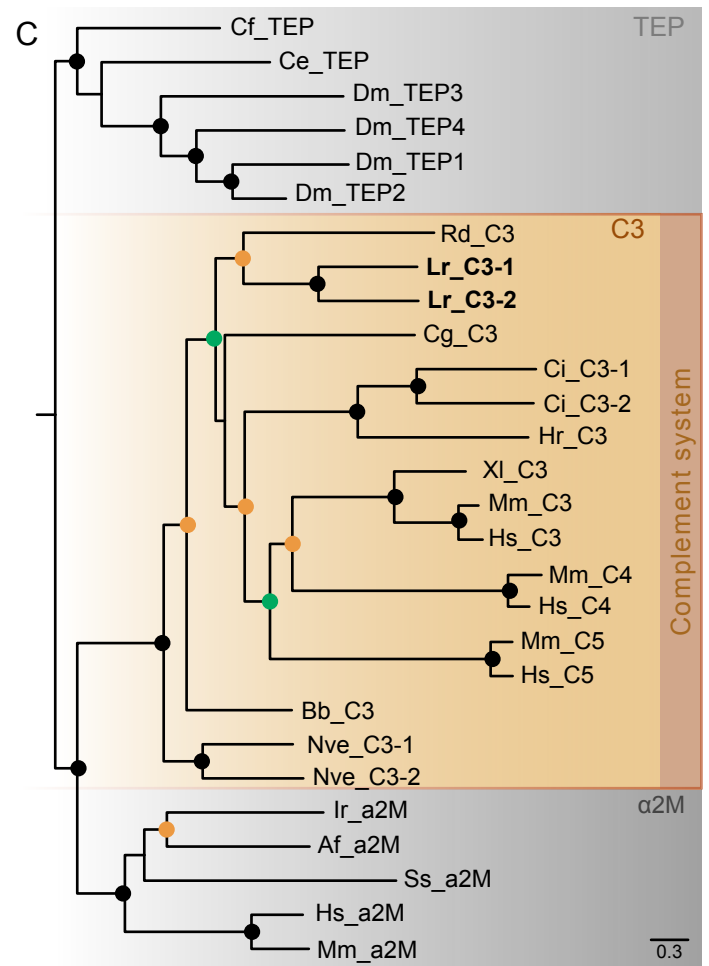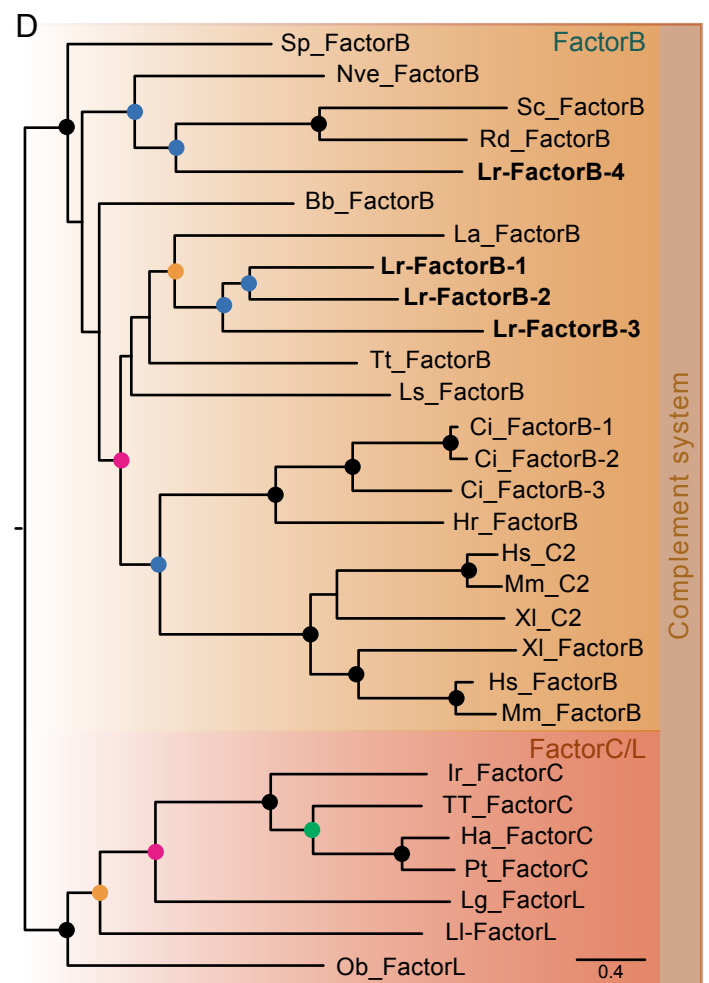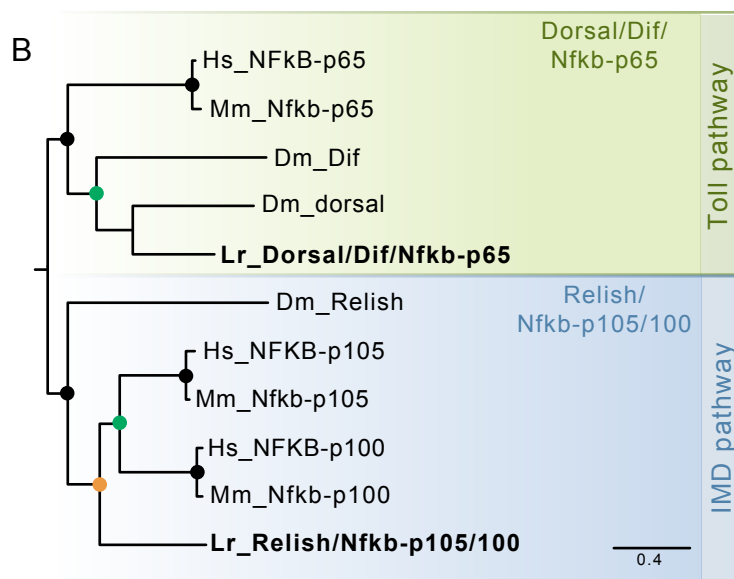

Supplement: Supplementary file 4 — Additional file 4: Fig. S1. Phylogenetic analyses of the putative components of the Toll-, the Imd- pathways and the complement system. A. Maximum likelihood phylogenetic analysis of DEATH domain containing proteins of the Toll and the Imd pathways. B. Maximum-likelihood phylogenetic analysis of Nfκb factors in Lineus ruber, Homo sapiens, Mus musculus and Drosophila melanogaster. C. Maximum-likelihood phylogenetic analysis of proteins belonging to the TEP family. TEP family is constituted by TEP, C3, and α2M proteins. D. Maximum-likelihood phylogenetic analysis of Factor B, C2, Factor C and Factor L proteins. For all trees, dots indicate support values ≥ 60 (black dots: 98-100%; blue dots: 90-97%; green dots: 80-89%; orange dots: 70-79%; pink dots: 60-69%). Tip labels indicate the species name abbreviation followed by the gene name. Lineus ruber proteins are labeled in bold. Species abbreviation: Af: Azumapecten farreri; Am: Apis mellifera; Bb: Branchiostoma belcheri; Ce: Caenorhabditis elegans; Cf: Chlamis farreri; Cg: Crassostrea gigas; Ci: Ciona intestinalis; Dm: Drosophila melanogaster; Ha: Hasarius adansoni; Hr: Halocynthia roretzi; Hs: Homo sapiens; Ir: Ixodes ricinus; La: Lingula anatina; Lg: Lottia gigantea; Ll: Littorina littorea; Lr: Lineus ruber; Ls: Lepidonotus squamatus; Mc: Mytilus coruscus; Mm: Mus musculus; Ms: Melanaphis sacchari; Nve: Nematostella vectensis; Nvi: Nasonia vitripennis; Ob: Octopus bimaculoides; Pt: Parasteatoda tepidariorum; Rd: Ruditapes decussatus; Sc: Sinonovacula constricta; Sd: Suberites domuncula; Spa: Scylla paramosain; Spu: Strongylocentrotus purpuratus; Ss: Scylla serrata; Tt: Tachypleus tridentatus; Xl: Xenopus laevis. [file 12915_2022_1482_MOESM4_ESM.pdf]

A

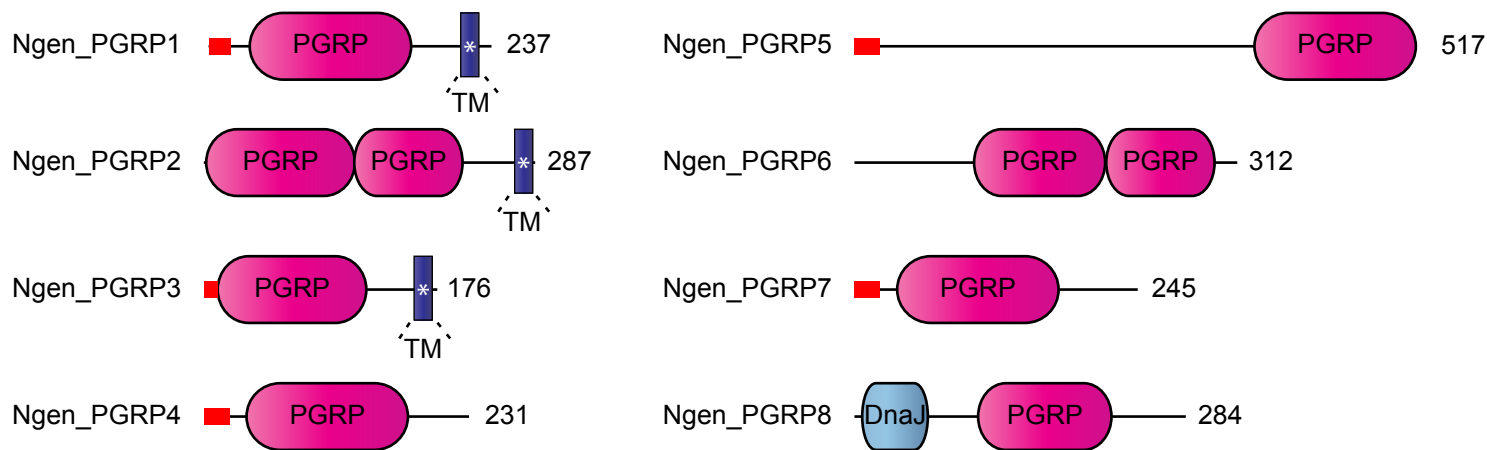

B

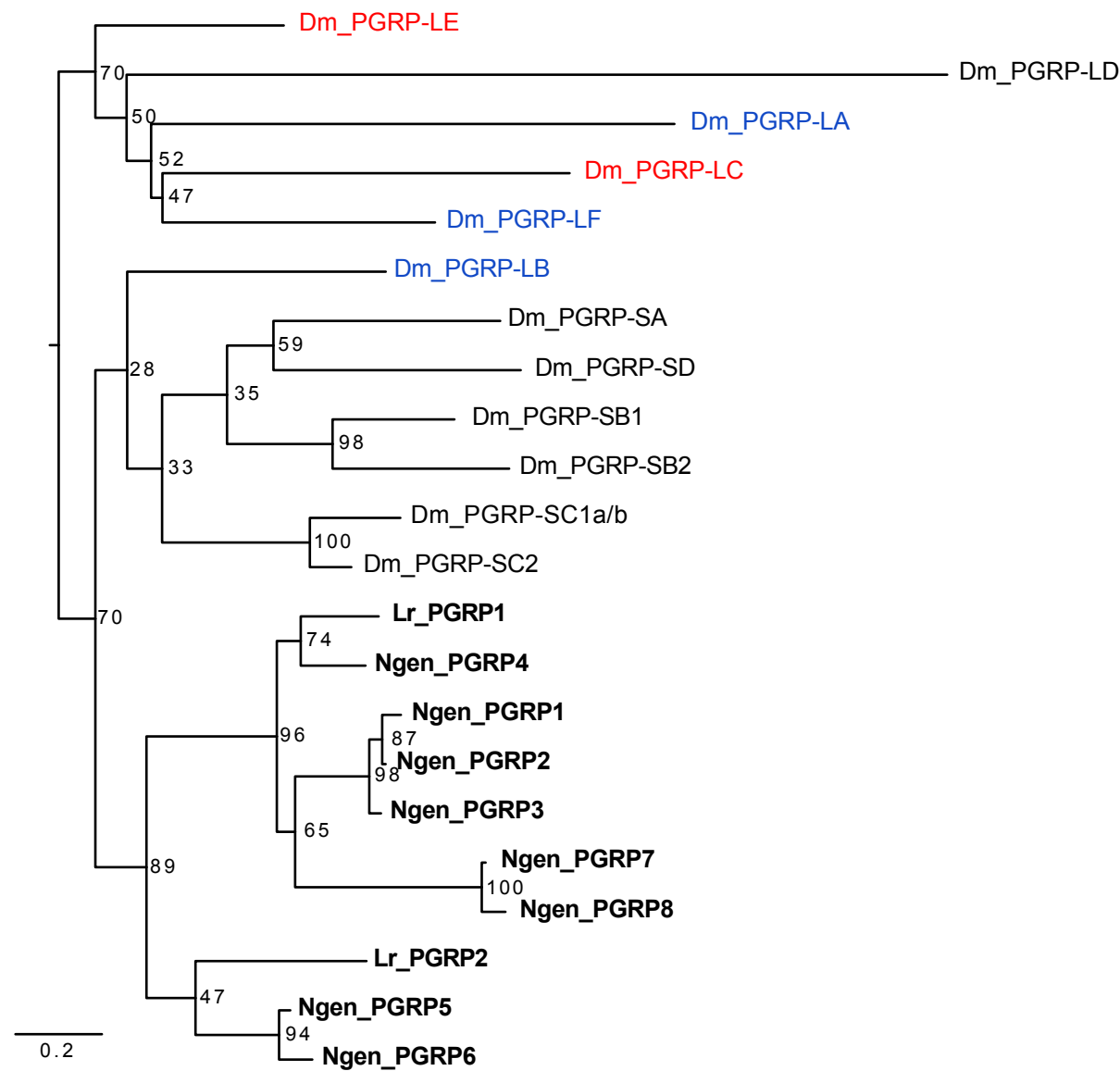

Supplement: Supplementary file 6 — Additional file 6: Fig. S3. PGRP proteins in Notospermus geniculatus and Lineus ruber. A. Domain architecture analyses of PGRPs in Notospermus geniculatus. All the PGRPs in Notospermus geniculatus contain only one PGRP domain, with the exception of Ngen_PGRP2 and Ngen_PGRP6. White asterisk indicates that transmembrane domains for Ngen_PGRP1-3 were only detected by hmmer online software and not SMART online software. Red rectangles indicate signal peptides. Numbers adjacent to each protein indicate the length of the protein in aminoacids. B. Maximum-likelihood phylogenetic analysis of PGRP proteins in Lineus ruber (Lr), Notospermus geniculatus (Ngen) and Drosophila melanogaster (Dm). Nemertean PGRP group forming an independent clade than Drosophila melanogaster PGRPs. Drosophila melanogaster sequences group forming two clades: a clade formed exclusively by long PGRPs and a clade formed by all short PGRPs and a non-transmembrane long PGRP (Dm_PGRP-LB). The later clade is the sister clade to the nemertean PGRPs. Numbers next to the tree nodes indicate bootstrap values. Red labels indicate Drosophila PGRPs involved in Imd pathway activation; Blue labels indicate Drosophila PGRPs involved in Imd pathway regulation. [file 12915_2022_1482_MOESM6_ESM.pdf]

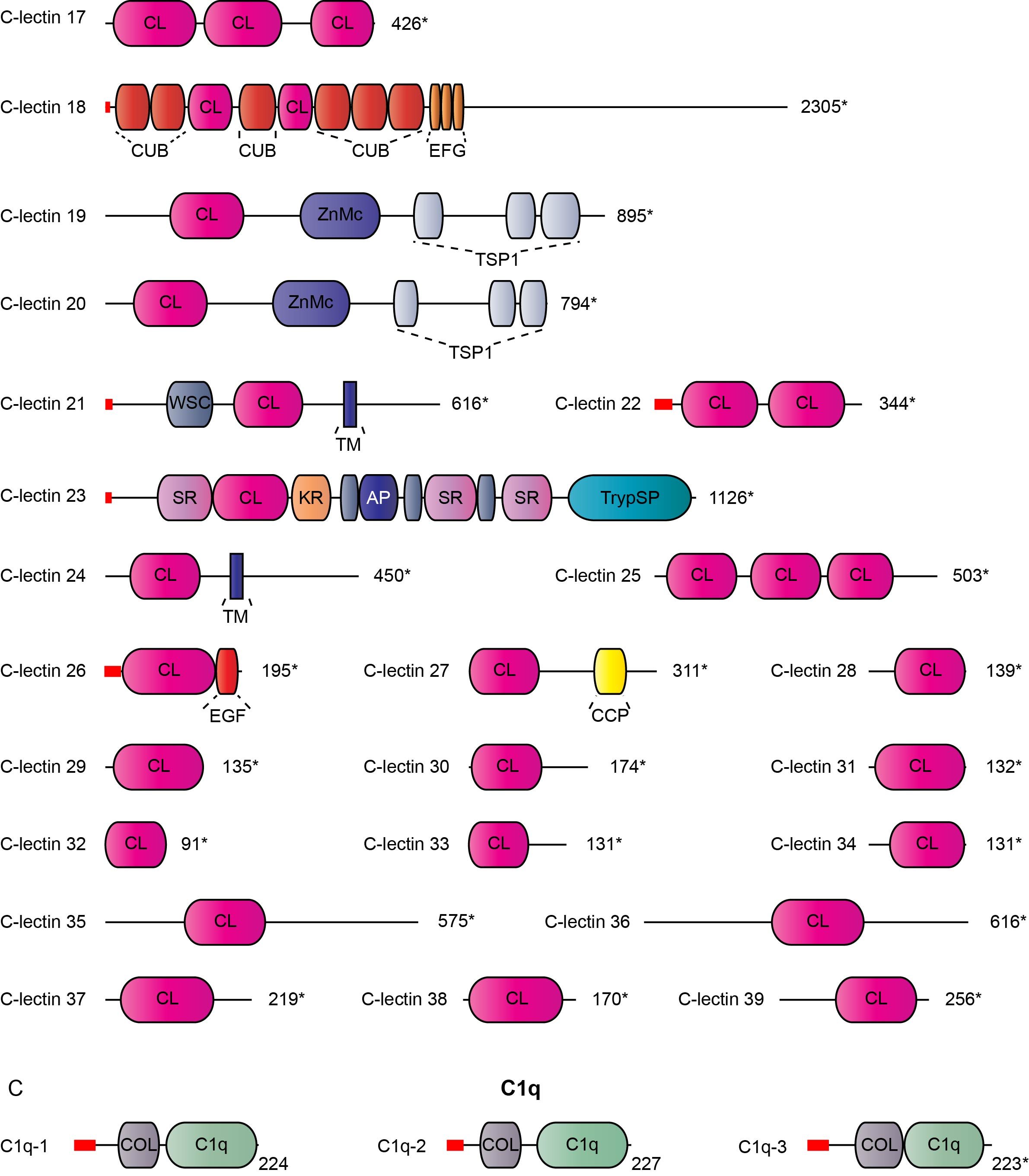

Supplement: Supplementary file 7 — Additional file 7: Fig. S4. FreD-C, lectins and C1q in Lineus ruber. A. Fibrinogen-related domain containing proteins (FreD-C). B. C-type lectins (C-lectins). C. C1q proteins. Numbers adjacent to each protein indicate the length of the protein in aminoacids. Asterisks next to the amino acid number indicate partial proteins. Red rectangles indicate signal peptides; blue small rectangles are coiled coils. [file 12915_2022_1482_MOESM7_ESM.docx]

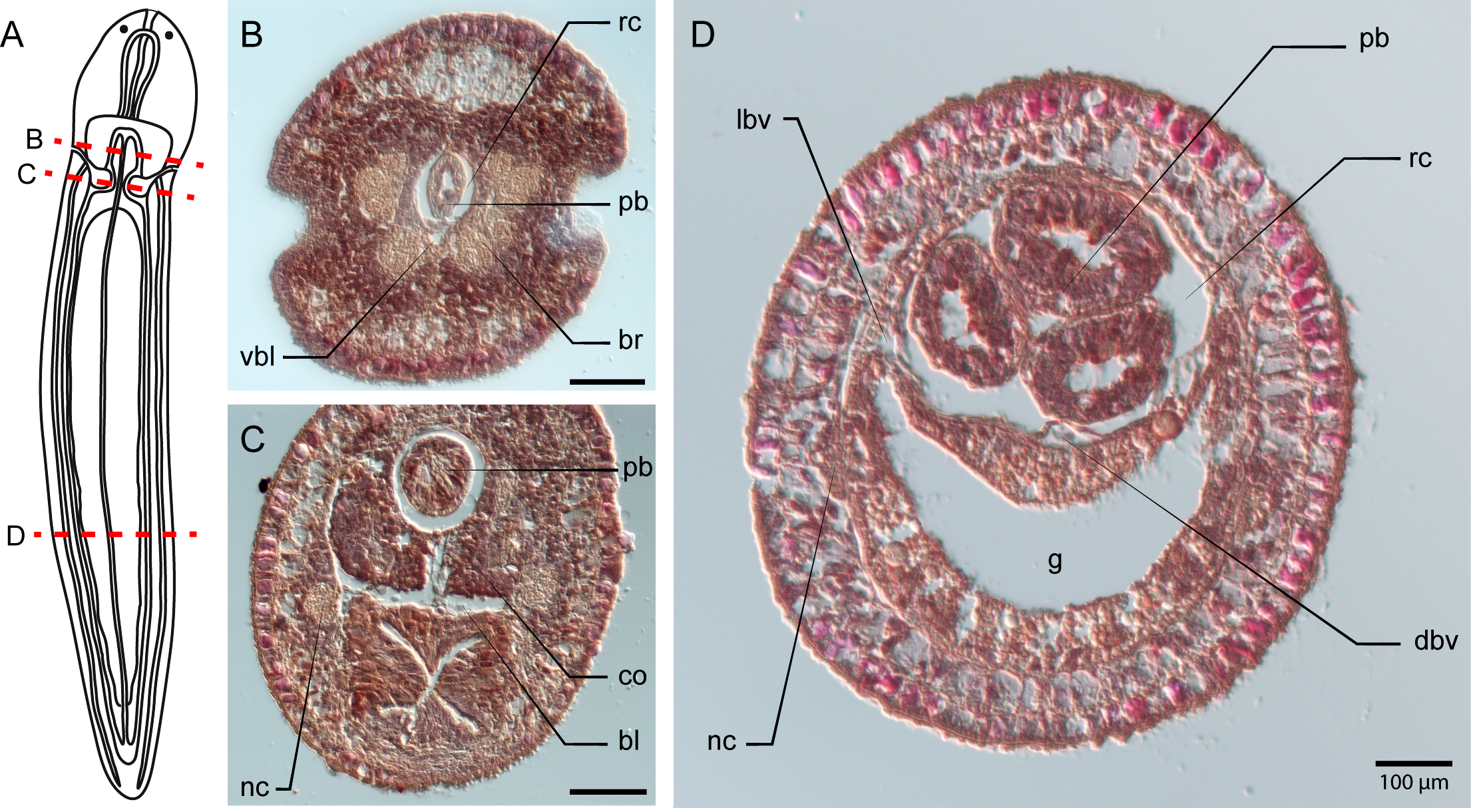

Supplement: Supplementary file 8 — Additional file 8: Fig. S5. Morphology of Lineus ruber juveniles. A. Diagram showing the level of the cross-sections on B-D panels. B-D. Hematoxilin-Eosin staining of cross-sections at different points across the anterior-posterior axis. Dorsal is to the top. All scale bars indicate 100μm. bl: blood lacunae; br: brain, co: cephalic organs; dbv: dorsal blood vessel; g: gut; lbv: lateral blood vessel; nc: nerve cord; pb: proboscis; rc: rhynchocoelum; vbl: ventral blood lacunae. [file 12915_2022_1482_MOESM8_ESM.docx]
