# Supplementary material for: The localization of Toll and Imd pathway and complement system components and their response to Vibrio infection in the nemertean Lineus ruber
Source: BMC Biol. 2023 Jan 12;21:7. doi: 10.1186/s12915-022-01482-1 (PMC9835746; doi:10.1186/s12915-022-01482-1)
Supplement: Supplementary file 9 — Additional file 9: Table S3. qPCR primers. [file 12915_2022_1482_MOESM9_ESM.docx]

**Additional file 9: Table S3 – qPCR primers**

| **Gene** | **Forward** | **Reverse** |
| --- | --- | --- |
| ***TLRα3*** | TCCTTTCCAATGTCACACACC | CAGTTCACTCCCACGAAGTTG |
| ***TLRα4*** | CACGGGGAAGACCAATCAATG | GGCGTTCATCCATACTCCAGTG |
| ***TLRβ1*** | CAACCAAACACGACTGTCAATGC | ACCACGAAACCCGCCTTTACTG |
| ***TLRβ2*** | GGTCGGTGCTAATGGACGATTC | CGCAATGGGTGTCAAACAGAC |
| ***imd*** | TGCTGGAAGTTGATTCAGTCGTC | GAGTAAGTTCACCAATGTCGCTACC |
| ***C3-1*** | AACTGAGGTTTGCGGACCACTG | CCCATCCTTTCCCATCACAAG |
| ***fred-c5*** | AGCGACAATGACCTCTGGTTTG | ATCCTGTTTCCACGAGTGCCAC |
| ***c-lectin2*** | GCAGGATGAAGCGAATGAAGAGAC | TTGGTTTCCCTTGCTCCACC |
