# Supplementary material for: The localization of Toll and Imd pathway and complement system components and their response to Vibrio infection in the nemertean Lineus ruber
Source: BMC Biol. 2023 Jan 12;21:7. doi: 10.1186/s12915-022-01482-1 (PMC9835746; doi:10.1186/s12915-022-01482-1)
Supplement: Supplementary file 11 — Additional file 11. Accession Numbers from sequences used for the phylogenetic analyses. [file 12915_2022_1482_MOESM11_ESM.docx]

Additional File Accession numbers:

Figure S2 Accession numbers: Dm_Imd: Q7K4Z4; Am_Imd: XP_016767530.2; Spa_Imd: AZK36044.1; Nvi_Imd: NP_001135910.1; Hs_MyD88: NP_001166037.2; Mm_MyD88: ID17874; Dm_MyD88: ID35956; Cg_MyD88: NP_001292287.1; La_MyD88: XP_013416180.1; Dm_Fadd: NP_651006.1; Hs_Fadd: NP_003815.1; Mm_Fadd: NP_034305.1; Cg_Fadd: NP_001295786.1; La_Fadd: XP_013392457.1; Dm_Tube: NP_001189164.1; Mm_Irak4: NP_084202.2; Hs_Irak4: NP_001107654.1; Cg_Irak4: XP_011428693.2; Ms_Irak4: XP_025204662.1; Dm_Pelle: NP_476971.1; Hs_Irak1: NP_001020413.1; Mm_Irak1: NP_001171444.1; Ci_Irak4: XP_009859368.1; Mm_Irak3: NP_001346113.1; Hs_Irak3: NP_001135995.1; Sd_Irak4-like: CAL36106.1; Hs_Irak2: NP_001561.3; Mm_Irak2: NP_001107025.1; Mc_Irak1: QCO95270.1; Dm_dorsal: P15330; Dm_Diff: P98149; Hs_NFkB-p65: Q04206; Mm_NFkB-p65: Q04207; Dm_Relish: Q94527; Hs_NFKB-p105: P19838; Mm_NFkB-p105: P25799; Hs_NFKB-p100: Q00653; Mm_Nfkb-p100: Q9WTK5; Dm_TEP1: CAB87807; Dm_TEP2: CAB87808; Dm_TEP3: CAB87809; Dm_TEP4: CAB87810; Cf_TEP: EF210036; Ce_TEP: CAB05007; Rd_C3: FJ392025; Cg_C3: NP_001292308.1 ; Nve_C3-1: AB450038; Nve_C3-2: AB450040; Ci_C3-1: Q8WPD8; Ci_C3-2: Q8WPD7; Hr_C3: AB006964; Bb_C3: AB050668; Xl_C3: AAB60608; Mm_C3: P01027; Hs_C3: P01024; Mm_C4: P01029; Hs_C4: AAB59537; Mm_C5: P06684; Hs_C5: P01031; Ir_a2M: EU835901; Hs_a2M: P01023; Mm_a2M: Q61838; Af_a2M: AAR39412; Ss_a2M: ABD61456; Hs_FactorB: CAA51389.1; Mm_FactorB: NP_001136178.1; Xl_FactorB: NP_001081234; Bb_FactorB: XP_019626103.1; Ci_FactorB-1: NP_001027973.1; Ci_FactorB-2: NP_001029011.1; Ci_FactorB-3: NP_001027974.1; Hr_FactorB: AAK00631; Spu_FactorB: NP_999700.1; La_FactorB: XP_013415956.1; Nve_FactorB: BAH22726.1; Sc_FactorB: QEX93860.1; Rd_FactorB: ACQ91095; Ls_FactorB: MG596914.1; Tt_FactorB: BAM15263; Hs_C2: NP_000054.2; Mm_C2: NP_038512.2; Xl_C2: NP_001116166.2; Ir_FactorC: AII02148.1; Tt_FactorC: P28175.1; Ha_FactorC: BAR45633.1; Pt_FactorC: XP_015930211.1; Lg_FactorL: XP_009058759; Ob_FactorL: XP_014774764; Ll_FactorL: MG596893.1.

*Figure S4: Drosophila melanogaster* accession numbers: Dm_PGRP-LA: Q95T64; Dm_PGRP-LD: Q9GN97; Dm_PGRP-LE: Q9VXN9; Dm_PGRP-LF: Q8SXQ7; Dm_PGRP-SA: Q9VYX7; Dm_PGRP-SB1: Q70PY2; Dm_PGRP-SB2: Q9VV96; Dm_PGRP-SC1a/b: C0HK98; Dm_PGRP-SC2: Q9V4X2; Dm_PGRP-SD: Q9VS97.
